# Oral/dental items in the resident assessment instrument – minimum Data Set 2.0 lack validity: results of a retrospective, longitudinal validation study

**DOI:** 10.1186/s12963-016-0108-y

**Published:** 2016-10-21

**Authors:** Matthias Hoben, Jeffrey W. Poss, Peter G. Norton, Carole A. Estabrooks

**Affiliations:** 1 Knowledge Utilization Studies Program (KUSP), Faculty of Nursing, University of Alberta, 5-006 Edmonton Clinic Health Academy (ECHA), 11405 87 Avenue, Edmonton, AB T6G 1C9 Canada; 2 School of Public Health and Health Systems, University of Waterloo, Waterloo, ON Canada; 3 Department of Family Medicine, University of Calgary, Calgary, AB Canada

**Keywords:** Dental care for aged, Quality improvement, Nursing homes, Outcome assessment (Health Care), Epidemiologic geriatric assessment, Psychometrics

## Abstract

**Background:**

Oral health in nursing home residents is poor. Robust, mandated assessment tools such as the Resident Assessment Instrument – Minimum Data Set (RAI-MDS) 2.0 are key to monitoring and improving quality of oral health care in nursing homes. However, psychometric properties of RAI-MDS 2.0 oral/dental items have been challenged and criterion validity of these items has never been assessed.

**Methods:**

We used 73,829 RAI-MDS 2.0 records (13,118 residents), collected in a stratified random sample of 30 urban nursing homes in Western Canada (2007–2012). We derived a subsample of all residents (*n* = 2,711) with an admission and two or more subsequent annual assessments. Using Generalized Estimating Equations, adjusted for known covariates of nursing home residents’ oral health, we assessed the association of oral/dental problems with time, dentate status, dementia, debris, and daily cleaning.

**Results:**

Prevalence of oral/dental problems fluctuated (4.8 %–5.6 %) with no significant differences across time. This range of prevalence is substantially smaller than the ones reported by studies using clinical assessments by dental professionals. Denture wearers were less likely than dentate residents to have oral/dental problems (adjusted odds ratio [OR] = 0.458, 95 % confidence interval [CI]: 0.308, 0.680). Residents lacking teeth and not wearing dentures had higher odds than dentate residents of oral/dental problems (adjusted OR = 2.718, 95 % CI: 1.845, 4.003). Oral/dental problems were more prevalent in persons with debris (OR = 2.187, 95 % CI: 1.565, 3.057). Of the other variables assessed, only age at assessment was significantly associated with oral/dental problems.

**Conclusions:**

Robust, reliable RAI-MDS 2.0 oral health indicators are vital to monitoring and improving oral health related quality and safety in nursing homes. However, severe underdetection of oral/dental problems and lack of association of well-known oral health predictors with oral/dental problems suggest validity problems. Lacking teeth and not wearing dentures should be considered an indicator for urgent oral/dental treatment needs.

**Electronic supplementary material:**

The online version of this article (doi:10.1186/s12963-016-0108-y) contains supplementary material, which is available to authorized users.

## Background

Oral health issues are among the 50 most prevalent pathologic conditions worldwide: dental caries of permanent teeth (rank 1, 35 %, *n* > 2.4 billion people), chronic periodontitis (rank 6, 11 %, > 740 million people), and edentulism (rank 36, 2 %, > 150 million people) [1]. In many instances oral health conditions are chronic conditions, sharing common, modifiable risk factors (e.g., tobacco use, excessive consumption of alcohol, unhealthy diet, stress, less than optimal personal hygiene) with other chronic diseases [2]. Periodontitis increases the risk for cardiovascular diseases, diabetes, or respiratory diseases [3], and periodontal diseases can be clinical manifestations of systemic diseases such as HIV, diabetes, respiratory diseases, and cancer [2]. Oral conditions can cause disability, and their global burden is comparable to conditions such as hypertensive heart disease, schizophrenia, anemias, and various forms of cancer [4].

Older people are at particular risk for poor oral health. As people age, their oral health deteriorates, partly through physical changes but primarily through reduced/limited access to dental services [5] and through chronic diseases that increase frailty and limit ability for self-care [6]. Therefore international organizations, such as the World Health Organization [7], the FDI World Dental Federation [8, 9], the English National Health System [10], the US Institutes of Medicine [11, 12], and the Canadian Academy of Health Sciences [5], have made strong policy statements calling for action to improve oral health care for frail older adults by applying an “oral-health-in-all-policies approach” [7], and proclaiming life-long oral health as a “fundamental human right” [7].

Oral health care is poor in nursing homes, internationally [13, 14] and in Canada [15, 16]. Nursing home residents are a highly vulnerable population at particularly high risk for poor oral health [5]. In agreement with international studies [17–19], one of the few Canadian studies on oral health of nursing home residents [16] found that 41 % of residents had no natural teeth, 41 % had mucosal abnormities, 5 % reported toothache, and 1 % reported severe tooth/mouth pain at night. Of residents with natural teeth, 51 % had untreated coronal caries and 44 % had untreated root caries.

Poor oral health raises health care costs and affects residents’ quality of life and safety through unnecessary pain, suffering, and elevated risk of malnutrition, aspiration pneumonia, respiratory diseases, diabetes, cardiovascular diseases, and premature death [3, 20–25]. Bad breath, changed dental aesthetics, and altered speech can affect self-image and self-esteem, with serious psychological and social consequences [26, 27].

By 2021, baby boomers will enter nursing homes in greater numbers with more of their natural teeth, more complex prostheses and bridges than previous generations, and significantly increased and different care needs [28]. Challenges in oral health care are further elevated by the rapidly growing number of residents with dementia, who need extra assistance and who may exhibit responsive behaviors that complicate care [29]. However, 80 % or more of direct care in Canadian nursing homes is provided by unregulated care aides with limited formal training who face challenging workloads [30, 31].

Improving oral health assessments in nursing homes is a priority to promote health and quality of life [32, 33] but the lack of mandated, robust tools is a major problem [5, 34, 35]. Most Canadian provinces mandate use of the Resident Assessment Instrument – Minimum Data Set (RAI-MDS) 2.0 [36] for nursing home residents on admission and subsequent quarterly intervals [37]. It is a valid, reliable standardized tool to assess residents’ clinical and functional characteristics [38, 39], which can effectively monitor and improve quality of care. The RAI-MDS 2.0 or the related interRAI Long-Term Care Facilities (LTCF) version is currently used in Europe (Belgium, England, Finland, France, Germany, Iceland, Italy, Netherlands, Norway, Spain, Sweden, Switzerland), Asia (Hong Kong, Korea, Japan), and the Pacific Rim (Australia, New Zealand) [40]. The United States is the only country using another related version, the RAI-MDS 3.0 [40]. The oral/dental items of the RAI-MDS 2.0 are completed only on the full assessment version, done on admission, and then annually or when significant change occurs. The quarterly version of the RAI-MDS 2.0 done at other three-month intervals omits the oral/dental items.

Reliability and validity literature for the RAI-MDS in general, and its oral health items in particular, go back to the instrument’s development more than 25 years ago [41, 42]. Reliability studies using interrater methods have generally reported strong findings for RAI-MDS 2.0 items [43–45] and quality indicators (QIs), defined as rates of clinically relevant outcomes, such as falls, pressure ulcers or pain, aggregated on unit or facility level [45] (kappa values > 0.7 for almost all items and QIs). Internal consistency reliability was found to be high for the Cognitive Performance Scale (CPS), depression and pain items [42]. Mor et al. [41], Poss et al. [38], and Shin and Scherer [42] provide summaries of the RAI-MDS 2.0 reliability and validity literature. The CPS and the Activities of Daily Living (ADL) scales especially were consistently found to have strong criterion and construct validity, while depression, behavior, pain and nutrition/weight loss items are less valid. Hirdes et al. [46] report internal consistency reliability and criterion validity results for the Canadian RAI-MDS 2.0 version. Cronbach’s alpha values for the ADL long form scale, the Depression Rating Scale (DRS) and the Aggressive Behaviour Scale (ABS) consistently exceeded 0.7, and criterion validity assessments demonstrated that cognition, ADL, continence, and behavior were related in the expected directions, with stable associations over time.

While the RAI-MDS 2.0 has been effectively used to monitor and improve safety and quality of care of nursing home residents [41, 47–49], early US studies indicate low reliability [43] and validity [20, 50–53] of the oral health components, partly due to items themselves and partly due to poorly trained assessors (care home staff) who underestimate mouth problems. Hawes et al. [43] report an average inter-rater reliability of 0.46 for the oral/dental items, which is at the low range of acceptable [54], and notably lower compared to other MDS items. Psychometric properties of the RAI oral health items are rarely reported, possibly because of their inclusion only on the full assessment version and their absence from important applications such as case mix or quality reporting. The American Dental Association (ADA) and Special Care Dentistry (SCD) challenged the content validity (i.e., completeness and appropriateness of wording) of the RAI-MDS 2.0 oral/dental items [20, 52]. Only 9 % of 236 surveyed nursing directors in US nursing homes thought the RAI oral/dental items were often useful to identify dental needs of residents [51]. Arvidson-Bufano [50], Folse [55], and Cohen-Mansfield [53] report severe underdetection of oral/dental problems by nursing home care staff using the RAI oral/dental items, compared to clinical assessments by dental professionals. However, Arvidson-Bufano [50] demonstrated that the assessment quality could be improved by a 30-min training of nursing home care staff.

Mandatory use of the RAI-MDS 2.0 in most Canadian nursing homes supports monitoring of multiple facilities and a large population of residents. However, the oral/dental items are rarely used for this purpose, in part because they are not reported on the quarterly assessment version, and to our knowledge publications assessing their criterion validity (relationship with other outcomes) are not available. Criterion validity is defined as the association of a measure with outcomes of interest, either concurrently or predictively, as expected based on theory, evidence, and common reasoning [56, 57]. The Standards for Educational and Psychological Testing [56] – recognized as best practice in developing and validating scientific assessment tools [57] – consider these analyses to be an important source of validity evidence. Our Translating Research in Elder Care (TREC) study [58] offers a large longitudinal data set that we exploited to assess the criterion validity of the RAI-MDS 2.0 oral/dental items. While comparisons of RAI-MDS 2.0 regular staff assessments with assessments done by dental professionals have been conducted, to the best of our knowledge our study is the first to systematically assess criterion validity of the RAI-MDS 2.0 oral health items, using a modeling-based approach to assess if oral/dental problems as measured by the RAI-MDS 2.0 are associated with predictors of oral health as expected.

## Methods

### Setting and sample

Our data were collected within the Translating Research in Elder Care (TREC) study (see [58] for details). The database included 73,829 RAI-MDS 2.0 records collected from 10,975 residents (2007–2012) from a stratified random sample of 30 urban nursing homes in Alberta, Saskatchewan, and Manitoba. We listed all nursing homes in the three provinces and stratified them by province, owner-operator model (private for-profit, public not-for-profit, voluntary not-for-profit), and size (small: <80 beds, medium: 80–120 beds, large: >120 beds). The 30 nursing homes randomly drawn from this population reflect the population proportionality of the three stratification criteria (for details, see [58]).

**Fig. 1:**
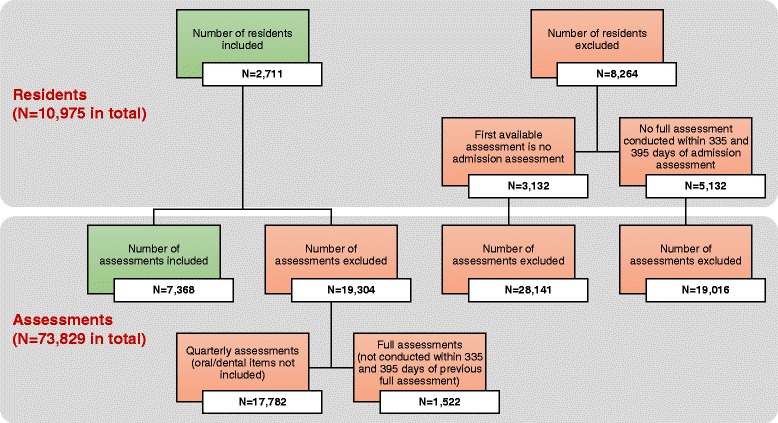
Overview of included and excluded residents and assessments

From our RAI-MDS 2.0 database, we selected all residents with an admission assessment plus one or more consecutive annual full assessments. This resulted in a study sample of 7,368 RAI-MDS 2.0 records from 2,711 residents (*n* = 1,471 residents with two full assessments, *n* = 703 residents with three full assessments, *n* = 376 residents with four full assessments, *n* = 153 residents with five full assessments, and *n* = 8 residents with six full assessments). Figure 1 details the number of residents and corresponding assessments excluded from our analyses, and the reasons for exclusion.

### Study design

This retrospective, longitudinal, and secondary data analysis assessed a) change in RAI-MDS 2.0 oral health variables over time and b) association of oral health variables with residents’ dentate status and with other variables known to influence residents’ oral health.

### Outcomes

#### Oral health variables

The RAI-MDS 2.0 full assessment [36] covers the following dichotomous oral/dental items:

1. Chewing problems (K1a)
2. Swallowing problems (K1b)
3. Mouth pain (K1c)
4. Debris (easily removable substances) in mouth at bedtime (L1a)
5. Dentures/removable bridge (L1b)
6. Some/all natural teeth lost, no dentures/partial plates available/used (L1c)
7. Broken, loose, or carious teeth (L1d)
8. Inflamed, swollen or bleeding gums; oral abscesses, ulcers, or rashes (L1e)
9. Daily oral health care by resident or staff (L1f)

#### Dependent variable

We combined the variables mouth pain (K1c), dental problems (L1d), and periodontal problems (L1e) into a dichotomous variable reflecting oral/dental problems overall (coded as 1 = one or more problems present or 0 = no problems present).

#### Independent variable

Our primary independent variable was *dentate status*, which is closely associated with oral health conditions (see Additional file 1 for a detailed description of the supporting evidence). We used RAI-MDS 2.0 variables L1b (dentures) and L1c (some/all teeth lost, no dentures used) to generate a categorical variable reflecting dentate status: *dentate* (“no” to L1b and L1c), *dentures* (“yes” to L1b, “no” to L1c), or *no dentures* (“yes” to L1c, regardless of the coding of L1b).

#### Covariates

Our model included RAI-MDS 2.0 variables (known association with oral/dental problems) of *dementia diagnosis* and *oral hygiene* (debris, daily cleaning). We adjusted our final model using covariates known to be associated with nursing home residents’ oral health (see Additional file 1 for details on the supporting evidence): *age at RAI-MDS 2.0 assessment* (continuous); *sex*; *physical functioning* (Activities of Daily Living – Hierarchical score [59] >3); *cognition* (Cognitive Performance Scale score [60] >3); *resists care at least weekly* (yes/no); *depressive symptoms* (Depression Rating Scale score [61] >2); and *dementia diagnosis*. We included *quarter of assessment* (e.g., Q4/2007) as a continuous covariate to adjust for potential systematic differences in assessment practices and awareness of oral/dental problems at different time points. All independent variables and covariates were used from the same assessment as the dependent variable.

### Statistical analyses

We used IBM® SPSS® version 23.0 for all analyses. With descriptive analyses, we compared prevalence of oral/dental problems and other resident characteristics over time. We ran General Estimating Equation (GEE) models to account for assessments nested within residents and to simultaneously assess effects of time, dentate status, and covariates on residents’ oral/dental problems. Model 1 included only dentate status as independent variable. Models 2–4 included dentate status plus dementia diagnosis, debris, or daily cleaning. Our final GEE model (model 5) included all those variables plus all additional covariates. We used a binomial logit link function with the dichotomous dependent variable and an autoregressive working correlation matrix (AR1) in all models. Due to the way RAI data are collected and cleaned in Canada, our data set did not include any missing variables. The completeness and integrity of RAI-MDS data items are extremely high in Canada due to universal use of electronic entry which constrains responses to available options, and only allows an assessment submission to occur when all items are completed. In addition, the Canadian Institute for Health Information, the national agency to which all facilities in TREC submit to, provides additional data checks on submitted records [37]. Therefore, the assumption underlying GEEs that data are missing completely at random (MCAR) was met on an individual item level. In addition, we checked if entire assessments (i.e., residents only having two as opposed to three to six assessments) were missing completely at random. We generated variables, indicating if the third, fourth, fifth, and sixth assessment were either missing = 0 or present = 1 at a specific time point. Using these variables, we performed Little’s MCAR test [62], which failed to reject the null hypothesis that data are MCAR (χ^2^ = 9.327, DF = 6, *P* = 0.156). In addition, using non-parametric exact tests for categorical outcomes, and t-tests for continuous outcomes, we compared study outcomes at baseline between residents with exactly two assessments and residents with three or more assessments (Additional file 2). Compared to residents with exactly two assessments, residents with three or more assessments were significantly less likely to resist care, to be highly physically impaired and depressive, more likely to be female, and their baseline assessments were conducted in later quarters. All other variables did not differ significantly.

We performed multicollinearity assessment of all covariates included in the final model. The results (Additional file 3) indicate no substantial multicollinearity with tolerance values clearly above the recognized threshold value of 0.1 [63]. We added variables in a stepwise approach. None of the regression coefficients or their related 95 % confidence interval (CI) changed substantially when adding or removing variables.

To check for possible model overfitting as well as for differences between residents included in the analyses and excluded residents, we conducted two types of sensitivity analysis. First, we ran four separate binary logistic regression models (corresponding to the final GEE model 5) with all first, second, third, and fourth assessments, respectively. Second, we compared admission information of our 2,711 included residents to admission information of all excluded residents with an admission assessment available (*n* = 5,132, Fig. 1), and we ran another binary logistic regression model (corresponding to the final GEE model 5), using the admission assessments of the excluded residents (Additional file 4).

As we used independent variables and covariates from the same assessment as the dependent variable, no conclusions are possible whether the independent variables/covariates (e.g., debris) predict oral/dental issues or if oral/dental issues predict the independent outcomes (reverse causality). Therefore, we ran the final model again, using independent outcomes/covariates from a) the assessment conducted previously to the one including the dependent variable, and b) the admission assessment of each resident (Additional file 5).

## Results

### Resident characteristics

Resident characteristics and prevalence of model variables changed over time of assessment (Table 1). Overall, residents became more physically dependent, cognitively impaired, and depressed, and resisted care more frequently. Rates of diabetes, atherosclerosis, and pneumonia remained roughly constant.

**Table 1:** Characteristics of included residents

	Assessment number					
	1	2	3	4	5	6
Sample size						
All residents (n or more assessments)	2711	2711	1240	537	161	8
Residents with exactly n assessments	NA	1471	703	376	153	8
Age assessment						
Age (Mean ± SD)	84.4 ± 8.9	85.4 ± 8.9	86.0 ± 8.8	86.6 ± 8.4	86.2 ± 8.7	82.3 ± 13.1
< 65 years	101 (3.7 %)	86 (3.2 %)	34 (2.7 %)	10 (1.9 %)	3 (1.9 %)	1 (12.5 %)
65–74 years	221 (8.2 %)	197 (7.3 %)	96 (7.7 %)	38 (7.1 %)	14 (8.7 %)	1 (12.5 %)
75–84 years	939 (34.6 %)	847 (31.2 %)	345 (27.8 %)	147 (27.4 %)	45 (28.0 %)	2 (25.0 %)
85–94 years	1220 (45.0 %)	1288 (47.5 %)	601 (48.5 %)	264 (49.2 %)	77 (47.8 %)	3 (37.5 %)
> 94 years	230 (8.5 %)	293 (10.8 %)	164 (13.2 %)	78 (14.5 %)	22 (13.7 %)	1 (12.5 %)
Sex						
Female	1849 (68.2 %)	1849 (68.2 %)	882 (71.1 %)	409 (76.2 %)	130 (80.7 %)	7 (87.5 %)
Physical functioning (Activities of Daily Living – Hierarchical (ADL-H) score)						
Independent (ADL-H < 2)	366 (13.5 %)	286 (0.5 %)	101 (8.1 %)	40 (7.4 %)	10 (6.2 %)	1 (12.5 %)
Medium dependent (ADL_H 2–4)	1775 (65.5 %)	1707 (63.0 %)	733 (59.1 %)	281 (52.3 %)	77 (47.8 %)	4 (50.0 %)
Highly dependent (ADL_H > 4)	570 (21.0 %)	718 (26.5 %)	406 (32.7 %)	216 (40.2 %)	74 (46.0 %)	3 (37.5 %)
Cognition (Cognitive Performance Scale (CPS) score)						
Relatively intact cognition (CPS < 2)	620 (22.9 %)	500 (18.4 %)	196 (15.8 %)	65 (12.1 %)	11 (6.8 %)	1 (12.5 %)
Mild/moderate impairment (CPS 2–3)	1440 (53.1 %)	1333 (49.2 %)	586 (47.3 %)	232 (43.2 %)	69 (42.9 %)	2 (25.0 %)
Severe impairment (CPS > 3)	651 (24.0 %)	878 (32.4 %)	458 (36.9 %)	240 (44.7 %)	81 (50.3 %)	5 (62.5 %)
Behavior/mood						
Resists care (at least once per week)	742 (27.4 %)	934 (34.5 %)	447 (36.0 %)	212 (39.5 %)	63 (39.1 %)	2 (25.0 %)
Depression Rating Scale score > 2	673 (24.8 %)	959 (35.4 %)	470 (37.9 %)	194 (36.1 %)	63 (39.1 %)	4 (50.0 %)
Medical diagnoses						
Dementia	1606 (59.2 %)	1813 (66.9 %)	856 (69.0 %)	393 (73.2 %)	120 (74.5 %)	6 (75.0 %)
Diabetes mellitus	522 (19.3 %)	533 (19.7 %)	256 (20.6 %)	97 (18.1 %)	30 (18.6 %)	1 (12.5 %)
Atherosclerotic heart disease	175 (5.8 %)	134 (4.9 %)	62 (5.0 %)	22 (4.1 %)	2 (1.2 %)	0 (0.0 %)
Pneumonia	42 (1.5 %)	36 (1.3 %)	12 (1.0 %)	5 (0.9 %)	2 (1.2 %)	0 (0.0 %)
Dentate status						
Dentate	754 (27.8 %)	528 (19.5 %)	182 (14.7 %)	64 (11.9 %)	19 (11.8 %)	0 (0.0 %)
Dentures	1502 (55.4 %)	1633 (60.2 %)	757 (61.0 %)	343 (63.9 %)	88 (54.7 %)	5 (62.5 %)
No dentures	455 (16.8 %)	550 (20.3 %)	301 (24.3 %)	130 (24.2 %)	54 (33.5 %)	3 (37.5 %)
Oral health						
No problems	2582 (95.2 %)	2572 (94.9 %)	1170 (94.4 %)	509 (94.8 %)	153 (95.0 %)	7 (87.5 %)
Tooth problems (L1d = yes)^a^	96 (3.5 %)	104 (3.8 %)	54 (4.4 %)	24 (4.5 %)	6 (3.7 %)	1 (12.5 %)
Periodontal problems (L1e = yes)^a^	26 (1.0 %)	31 (1.1 %)	15 (1.2 %)	6 (1.1 %)	3 (1.9 %)	1 (12.5 %)
Mouth pain (K1c = yes)^a^	28 (1.0 %)	21 (0.8 %)	9 (0.7 %)	5 (0.9 %)	2 (1.2 %)	1 (12.5 %)
Any oral/dental problem^b^	129 (4.8 %)	139 (5.1 %)	70 (5.6 %)	28 (5.2 %)	8 (5.0 %)	1 (12.5 %)
Chewing problem (K1a = yes)	355 (13.1 %)	480 (17.7 %)	295 (23.8 %)	134 (25.0 %)	52 (32.3 %)	3 (37.5 %)
Swallowing problem (K1b = yes)	324 (12.0 %)	425 (15.7 %)	225 (18.1 %)	103 (19.2 %)	40 (24.8 %)	3 (37.5 %)
Debris (L1a = yes)	284 (10.5 %)	317 (11.7 %)	140 (11.3 %)	64 (11.9 %)	24 (14.9 %)	1 (12.5 %)
No daily cleaning (L1f = no)	171 (6.3 %)	103 (3.8 %)	33 (2.7 %)	10 (1.9 %)	1 (0.6 %)	0 (0.0 %)

^a^Sum of Residents with L1d = yes, L1e = yes and K1c = yes may be bigger than the number of residents with oral/dental issues, as residents may have more than on oral/dental problem

^b^Count of residents who had one or more of tooth problems, periodontal problems, or mouth pain

### Dentate status

Numbers of fully dentate residents decreased substantially over time (Table 1). Residents lacking teeth (edentulous) but not wearing dentures, the smallest group at first assessment, outnumbered dentate residents by the second assessment.

### Oral/dental problems

**Fig. 2:**
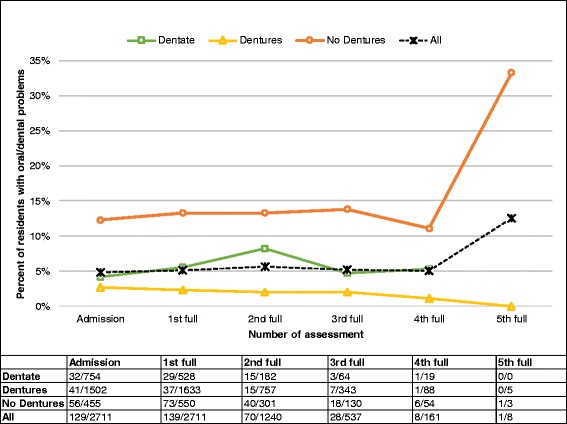
Oral/dental problems by dentate status over time. Legend: Number in the table represent number of residents with oral/dental problem/total number of residents

Tooth problems, periodontal problems, and mouth pain fluctuated across assessments (Table 1). Almost all residents (87.5 %–95.2 %) were assessed with no oral health problems. In all GEE models, dentate status was strongly associated with oral/dental problems (Table 2, Fig. 2). The final adjusted model, including all covariates, indicated that the odds to have oral/dental problems were less than half as high for denture wearers than for dentate residents. Edentulous residents not wearing dentures had almost two times higher odds of oral/dental problems than dentate residents. Oral debris and age at assessment were associated at statistically significant levels with oral/dental problems. Dementia and cognition care resistant behavior, physical functioning, depression, daily cleaning, and female sex were not significant predictors of oral health overall. Oral/dental problems of individual residents did not change significantly over time.

**Table 2:** General estimating equation models of outcomes related to oral/dental problems (*N* = 2,711 residents)

	Model 1			Model 2			Model 3			Model 4			Model 5		
Parameter	Est.	95 % CI	P	Est.	95 % CI	P	Est.	95 % CI	P	Est.	95 % CI	P	Est.	95 % CI	P
Assessment 1	Reference			Reference			Reference			Reference			Reference		
Assessment 2	1.053	0.878–1.264	0.576	1.061	0.883–1.274	0.582	1.041	0.866–1.251	0.668	1.049	0.874–1.259	0.609	0.971	0.779–1.211	0.792
Assessment 3	1.102	0.835–1.455	0.492	1.112	0.841–1.470	0.458	1.083	0.816–1.436	0.581	1.096	0.830–1.447	0.518	0.991	0.685–1.433	0.962
Assessment 4	1.028	0.677–1.562	0.896	1.042	0.688–1.577	0.848	1.020	0.670–1.554	0.925	1.023	0.673–1.553	0.917	0.921	0.545–1.558	0.759
Assessment 5	0.807	0.384–1.696	0.571	0.814	0.338–1.710	0.587	0.755	0.359–1.591	0.461	0.798	0.379–1.678	0.552	0.637	0.272–1.492	0.299
Assessment 6	2.307	0.294–18.085	0.426	2.329	0.292–18.588	0.425	2.085	0.348–12.483	0.421	2.282	0.291–17.897	0.433	1.712	0.254–11.531	0.581
Dentate	Reference			Reference			Reference			Reference			Reference		
Dentures	**0.434**	**0.295–0.641**	**<0.001**	**0.435**	**0.295–0.642**	**<0.001**	**0.433**	**0.294–0.638**	**<0.001**	**0.435**	**0.295–0.641**	**<0.001**	**0.458**	**0.308–0.680**	**<0.001**
No Dentures	**2.716**	**1.859–3.968**	**<0.001**	**2.725**	**1.865–3.983**	**<0.001**	**2.672**	**1.827–3.909**	**<0.001**	**2.726**	**1.864–3.988**	**<0.001**	**2.718**	**1.845–4.003**	**<0.001**
Dementia diagnosis	―	―	―	0.913	0.682–1.221	0.538	―	―	―	―	―	―	0.961	0.700–1.318	0.803
Debris	―	―	―	―	―	―	**2.361**	**1.693–3.294**	**<0.001**	―	―	―	**2.187**	**1.565–3.057**	**<0.001**
Daily cleaning	―	―	―	―	―	―	―	―	―	1.164	0.656–2.068	0.603	1.123	0.637–1.979	0.688
Female	―	―	―	―	―	―	―	―	―	―	―	―	0.871	0.644–1.179	0.371
Age at assessment^a^	―	―	―	―	―	―	―	―	―	―	―	―	**0.983**	**0.969–0.998**	**0.031**
CPS score > 3	―	―	―	―	―	―	―	―	―	―	―	―	0.842	0.611–1.159	0.292
ADL-H score > 3	―	―	―	―	―	―	―	―	―	―	―	―	1.160	0.867–1.551	0.318
Resists care	―	―	―	―	―	―	―	―	―	―	―	―	1.216	0.926–1.596	0.159
DRS score > 2	―	―	―	―	―	―	―	―	―	―	―	―	1.104	0.840–1.450	0.478
Assessment quarter^a^	―	―	―	―	―	―	―	―	―	―	―	―	1.016	0.981–1.052	0.377

Bold = statistically significant (*p* < .05)

*Est*. Parameter estimate (odds ratios for the independent variables), *CI* Confidence Interval, *CPS* Cognitive Performance Scale, *ADL-H* Activities of Daily Living – Hierarchical Scale, *DRS* Depression Rating Scale

^a^Continuous variable

### Sensitivity analysis

The cohort of excluded residents with an admission assessment did not differ from our included residents with respect to age, dementia diagnosis, atherosclerotic heart disease, and oral/dental problems (Table 3). However, excluded residents were more likely to be male, more physically dependent, cognitively impaired, depressed, resisted care more frequently, and more often had diabetes or pneumonia than our study cohort. Also, excluded residents wore dentures less often, had more chewing and swallowing problems, debris, and received daily cleaning more frequently than our study cohort. Despite these substantial differences, the binary logistic regression model conducted with the excluded residents, as well as the other regression models support the findings of our GEE models (Additional file 4). The additional GEE models, using independent outcomes/covariates from a) the assessment conducted previously to the one including the dependent variable, and b) the admission assessment of each resident, also confirm our conclusions (Additional file 5). In addition, they support the assumption that the independent outcomes indeed precede the dependent variable (oral/dental issues).

**Table 3:** Comparison of characteristics of included and excluded residents

	Study cohort	Excluded cohort	*P*
Sample size	2711	5132	NA
Age assessment			
Age (Mean ± SD)	84.4 ± 8.9	84.6 ± 9.0	0.266
Sex			
**Female**	**1849 (68.2 %)**	**3078 (60.0 %)**	**<0.001**
Functional abilities			
**Activities of Daily Living – Hierarchical (ADL-H) score > 3**	**867 (32.0 %)**	**2180 (42.5 %)**	**<0.001**
**Cognitive Performance Scale (CPS) score > 3**	**651 (24.0 %)**	**1450 (28.3 %)**	**<0.001**
Behavior/mood			
**Resists care (at least once per week)**	**742 (27.4 %)**	**1572 (30.6 %)**	**0.003**
**Depression Rating Scale score > 2**	**673 (24.8 %)**	**1450 (28.3 %)**	**0.001**
Medical diagnoses			
Dementia	1606 (59.2 %)	3067 (59.8 %)	0.663
**Diabetes mellitus**	**522 (19.3 %)**	**1099 (21.4 %)**	**0.026**
Atherosclerotic heart disease	175 (5.8 %)	305 5.9 %)	0.801
**Pneumonia**	**42 (1.5 %)**	**116 (2.3 %)**	**0.035**
Dentate status			
Dentate	754 (27.8 %)	1474 (28.7 %)	0.400
**Dentures**	**1502 (55.4 %)**	**2648 (51.6 %)**	**0.001**
**No dentures**	**455 (16.8 %)**	**1010 (19.7 %)**	**0.002**
Oral health			
Tooth problems (L1d = yes)^a^	96 (3.5 %)	194 (3.8 %)	0.615
Periodontal problems (L1e = yes)^a^	26 (1.0 %)	66 (1.3 %)	0.226
Mouth pain (K1c = yes)^a^	28 (1.0 %)	60 (1.2 %)	0.653
Any oral/dental problem^b^	129 (4.8 %)	282 (5.5 %)	0.166
**Chewing problem (K1a = yes)**	**355 (13.1 %)**	**880 (17.1 %)**	**<0.001**
**Swallowing problem (K1b = yes)**	**324 (12.0 %)**	**866 (19.9 %)**	**<0.001**
**Debris (L1a = yes)**	**284 (10.5 %)**	**752 (14.7 %)**	**<0.001**
**No daily cleaning (L1f = no)**	**171 (6.3 %)**	**228 (4.4 %)**	**<0.001**

Bold = statistically significant (*p* < .05)

^a^Sum of Residents with L1d = yes, L1e = yes and K1c = yes may be bigger than the number of residents with oral/dental issues, as residents may have more than on oral/dental problem

^b^Count of residents who had one or more of tooth problems, periodontal problems, or mouth pain

## Discussion

We measured variation of nursing home residents’ oral/dental problems as assessed by the RAI-MDS 2.0 oral/dental items over time, and association of well-known covariates of nursing home residents’ oral health with those oral health problems. Our findings indicate validity problems for the Canadian RAI-MDS 2.0 oral/dental items.

### Prevalence of oral/dental problems

Our prevalence findings are similar to other studies using RAI-MDS 2.0 oral/dental items [53, 64–66]: tooth problems (our study, 3.5 %–4.5 %; other studies, 3.0 %–7.7 %); periodontal problems (our study, 1.0 %–1.9 %; other studies, 0 %–5.4 %); mouth pain (our study, 0.7 %–1.2 %; other studies, 0 %–9.5 %); and overall oral/dental problems (our study, 4.8 %–5.6 %; one other study, 7.6 % [67]).

However, comparing our findings with studies using clinical assessments by dental professionals indicates that – like the US version of the RAI-MDS 2.0 [53, 55, 68] – the RAI-MDS 2.0 in our study significantly underdetects oral/dental problems. Clinical assessments find caries rates of 44.4 %–76.2 % among dentate residents [16–19, 69–71]. Among all residents, 31.7 %–48.7 % need periodontal treatment [16, 70, 72], 65.6 %–74 % have gingivitis [16, 19], 33.3 %–41.2 % have mucosal abnormalities [16, 71], 5.2 %–8.2 % have dental pain [16, 72], 1.2 % have severe tooth/mouth pain at night [16], and 3.4 % report gum pain or discomfort [70].

### Association of oral/dental problems with other variables

According to the literature, each variable in our model is robustly associated with nursing home residents’ oral health (Additional file 1). However, we saw significant association of only a few included variables with oral/dental problems, further underscoring potential validity problems with the Canadian RAI-MDS 2.0 oral/dental items. We did find three significant predictors of oral/dental problems in our models: dentate status, debris, and age at assessment.

#### Dentate status

Our measured dentate status rates are similar to other studies. In this study, 54.7 %–63.9 % of residents wore dentures, comparable to 45.9 %–57.5 % in other studies based on RAI-MDS 2.0 data [53, 64, 65]. Studies using different tools or methods [73, 74] found that 24.4 %–43.6 % of residents wore complete dentures and 25 %–26.9 % wore removable partial dentures. Matthews et al. [16] reported 68.1 % of residents with maxillary (upper) dentures and 35 % with mandibular (lower) dentures. In our study, 18.5 %–23.3 % of residents lacking some or all natural teeth did not wear dentures, compared to 14.8 %–35 % in studies using RAI-MDS 2.0 data [64–66] and 36 % in Matthews et al. [16].

A third to a half of residents in North American nursing homes lack teeth [16, 19, 71, 75, 76], the source of potential dental problems and most oral/dental problems. This fits our finding that denture wearers have fewer oral/dental problems. In contrast, denture wearers show higher risk of periodontal problems than dentate older adults [22, 77], a finding not confirmed by our study. Nursing home staff, who complete the RAI-MDS 2.0, may not detect periodontal problems; staff are not well-trained in oral health assessments and oral health care is a neglected topic in nursing homes [35, 78–82].

Our study confirms that tooth loss is associated with oral health problems in older adults who do not wear dentures [22, 77]. Residents may be discouraged from wearing dentures by painful remaining teeth, gum diseases such as gingivitis or periodontitis, or irritated gum tissues from ill-fitting dentures. Matthews et al. [16] found that almost 60 % of residents’ dentures were ill-fitting.

#### Oral debris

Poor oral hygiene is a major risk factor for tooth loss as well as periodontal diseases, and maintaining good oral hygiene helps retaining teeth and preventing oral inflammatory processes [83, 84]. Matthews et al. [16] found that a high debris score (≥2) increased the risk for coronal decay (adjusted OR = 2.12, 95 % CI: 1.02, 4.34). This matches the results of our final model in which oral debris was significantly associated with oral/dental problems (OR = 2.187, 95 % CI: 1.565, 3.057).

#### Age at assessment

One of the strongest risk factors for poor oral health is old age – partly due to physical changes, but primarily due to frailty, multiple chronic diseases increasing self-care deficiencies, and higher barriers to accessing professional dental services [85, 86]. While we found that age at assessment was significantly associated with oral/dental problems in our final model, our findings suggest that younger residents are more likely to have oral/dental problems than older residents. Specifically, the odds of oral/dental problems decrease by 1.7 % with each additional year of age (OR = 0.983 in our model) or by 8.2 % with each additional 5 years of age (OR = 0.983^5^ = 0.918). This is in disagreement with the available evidence, and it may be another indicator of validity problems related to the RAI-MDS 2.0 oral/dental items. However, the available evidence mostly focuses on older adults in general. Specific evidence on the effect of age on nursing home residents’ oral health is limited (see [16] as one of the few examples suggesting that older nursing home residents have poorer oral health than younger nursing home residents). Therefore, another possible reason for our finding may be that people whose health and personal situations require a move into a nursing home at a younger age may have generally poorer oral health than people who are older when moving into the facility. There is some evidence (although weak and inconsistent) that availability of strong social networks, especially of a spouse, partner, or friend who is able to assist with the necessary care can prevent a nursing home admission [87]. Although the supporting evidence is inconclusive [88], older people are thought to be more vulnerable to loneliness and social isolation than younger people, due to the loss of spouses, partners, and other confiding relationships [89]. Therefore, strong informal supports that protect from nursing home admission may be absent at older age resulting in relatively better health as well as functional and oral health status at nursing home admission, compared to admission at younger age. Furthermore, younger people who move into a nursing home may have different disease processes which might be associated with different risks for oral/dental problems than the typical frail senior, but further studies would be needed to establish the validity of these assumptions.

#### Change over time

Although oral/dental problems did not significantly change for individual residents across the six assessments, they increased overall in our sample. In quarters 3/2007, 4/2007, and 2/2008, 0 % of records indicated oral/dental problems; in quarter 2/2012, 12.7 % of records indicated problems. With our data, we cannot determine whether this increase reflects true trends for Canadian nursing home residents over time or increased detection of problems by care providers completing assessments. The age-standardized prevalence of untreated caries [90] and severe periodontitis [91] remained constant in North America between 1990 and 2010; prevalence of severe tooth loss decreased [92]. However, evidence is scarce, and often of poor quality, on trends over time for older adults’ oral health [93, 94]. Even less is known about nursing home residents’ oral health over time. In 2007 the RAI-MDS 2.0 had been implemented only recently in Alberta, Saskatchewan, and Manitoba, with substantial later efforts to improve data quality [37, 95–97]. Most likely, care providers completing assessments became better at detecting oral/dental problems.

The interRAI LTCF is poised to be the successor of the RAI-MDS 2.0 in Canada. It drops or modifies some of the oral/dental items and does not use an abbreviated quarterly version omitting these items, and thus may be better suited for monitoring quality in this area, compared to the RAI-MDS 2.0.

### Strengths and limitations

#### Strengths

Our study is the first to assess criterion validity of oral/dental items in the RAI-MDS 2.0 and to assess them over time (five consecutive years). It is unique in assessing differences in rates of oral/dental problems by resident dentate status. We used GEE models that are robust against non-normality, do not require continuous data, account for dependency of multiple assessments per resident, and simultaneously assess effects of various factors. From a representative sample of residents in Western Canadian nursing homes, we included all residents with an admission assessment plus two or more consecutive annual full assessments.

#### Limitations

We excluded all residents with no admission assessment available and all residents with an admission assessment but no consecutive annual assessment available. Our cohort may therefore not represent the population of short-stay nursing home residents with a length of stay of less than 12 months [98]. They might be healthier and have lower prevalence of oral/dental problems. When comparing our sample to excluded residents with admission assessments available, we noticed that the excluded residents are sicker and frailer than our study sample, but do not differ with respect to oral/dental problems, and a binary logistic regression using admission assessments of excluded residents confirmed results of our GEE model. As we were interested in general associations between variables rather than in causal relationships and generalizable effect sizes, we decided not to include sampling weights (i.e., proportional representation of residents according to the strata of the stratified random sample) into our models. Including these sampling weights may have altered our findings [99]. Also, the assumption relevant to GEEs that data are missing completely at random may not have been met entirely in our data set. We did not have any missing items, and Little’s MCAR test was non-significant for missed follow-up assessments. But residents with exactly two assessments differed in some baseline outcomes from residents with three or more assessments. However, prevalence of oral/dental problems in our study was similar to other studies using RAI-MDS 2.0 data, indicating that such data generally underdetect oral/dental problems. RAI-MDS variables do not allow detailed assessment of kinds of tooth or periodontal problems or of dentate status. Information was missing on how many residents are completely edentulous or have some teeth left, type of dentures used (complete, partial, upper, lower), and why residents do not wear dentures. Further studies based on detailed clinical assessments could determine if specific oral health problems are associated with specific dental and prostheses status.

## Conclusions

Oral health in nursing home residents is poor. Compared to non-institutionalized older adults, nursing home residents are particularly frail and barriers to accessing dental services are even higher. Care providers should pay special attention to oral health of edentulous residents not wearing dentures.

Oral health of nursing home residents must be assessed regularly using a valid, reliable, and practical tool. RAI-MDS 2.0 oral/dental items likely significantly underdetect oral/dental problems and are not associated with well-proven predictors for oral health, indicating poor validity [57]. Research is needed on whether these validity problems are due to inappropriate application of the tool or if the tool is flawed more fundamentally (i.e., vague and poorly defined constructs). In the former case, major investments into additional training of assessors and modified prioritizing are required, in the latter case, RAI-MDS 2.0 oral/dental items need to be modified or supplemented by more robust tools. The potential effect of the interRAI LTCF with its modified oral/dental items and more frequent collection is unknown. Residents’ dentate status is a promising indicator of poor oral health and as such, a promising avenue for improving quality of care and quality of life.

Widespread use and the longitudinal nature of the RAI-MDS 2.0 offer great potential to close a severe knowledge gap in nursing home residents’ oral health: trajectories of oral health problems for residents, health regions, and jurisdictions. By incorporating robust items with acceptable reliability and validity, the RAI-MDS 2.0 can also be used to monitor and improve quality and safety of oral health care in nursing homes.

## Additional files

Additional file 1: Justification for included independent variables/covariates. (PDF 124 kb)
Additional file 2: Comparison of baseline outcomes between residents with exactly two assessments and residents with three or more assessments. (PDF 16 kb)
Additional file 3: Results of the multicollinearity assessment. (PDF 191 kb)
Additional file 4: Results of the binary logistic regression models. (PDF 36 kb)
Additional file 5: Additional General Estimating Equations (final, fully adjusted model), using variables of a) the assessment conducted previously to the assessment including the oral/dental issues variable and b) the admission assessment. (PDF 19 kb)
