# Supplementary material for: Oral/dental items in the resident assessment instrument – minimum Data Set 2.0 lack validity: results of a retrospective, longitudinal validation study
Source: Popul Health Metr. 2016 Oct 21;14:36. doi: 10.1186/s12963-016-0108-y (PMC5073836; doi:10.1186/s12963-016-0108-y)
Supplement: Additional file 1: — Justification for included independent variables/covariates. (PDF 124 kb) [file 12963_2016_108_MOESM1_ESM.pdf]

ADDITIONAL FILE 1

Justification for included independent variables/covariates

Dentate status

Caries and periodontal diseases are the primary reasons for tooth loss [1, 2], leading to changes of normal physiology, impaired chewing functions, and nutritional problems [3]. Being without teeth decreases regeneration and resistance of oral tissues and impairs protective function of the oral mucosa [3]. Wearing partial removable dentures increases risk for plaque, gingivitis, and caries [4], and removable prostheses are predictors for multiple tooth loss in older adults [5]. Denture use is also associated with inflammatory oral mucosal disorders and traumatic ulcers [3]. Hence tooth loss is related to significantly decreased oral-health-related quality of life [6, 7].

Dementia

Persons with dementia have generally poorer oral health compared to the general population, including higher rates of dental cries, edentulism, and periodontal diseases [8]. Lacking self-care abilities including carrying out oral hygiene care, difficulties in wearing dentures, and poor oral hygiene are the assumed causes of poor oral health in persons with dementia. Care providers often lack training in proper oral health care as well as in managing residents with responsive behaviors, and they often lack awareness of the importance of appropriate oral hygiene [8]. In addition there is emerging evidence that poor oral health is also a risk factor for dementia [9]. The inflammatory processes that go along with periodontal diseases may be an important factor contributing to

impaired cognition, and masticatory inefficiencies caused by tooth loss may lead to dietary deficiencies associated with cognitive impairment.

Oral hygiene

Poor oral hygiene is a major risk factor for tooth loss as well as periodontal diseases, and maintaining good oral hygiene helps retaining teeth and preventing oral inflammatory processes [1, 2].

Age

One of the strongest risk factors for poor oral health is old age – partly due to physical changes, but primarily due to frailty, multiple chronic diseases increasing self-care deficiencies, and higher barriers to accessing professional dental services [10, 11].

Sex

Women have generally poorer oral health than men, due to biologic reasons (different salivary composition and flow rate, hormonal fluctuations, genetic variations), cultural factors (social roles), different barriers in accessing dental services, and dietary variations [12-14].

Physical functioning

Higher dependency levels in carrying out basic activities of daily living (including performing oral hygiene) is associated with poor oral health [8, 15].

Responsive behaviors

One of the major barriers nursing home care providers encounter when providing oral health care to residents is residents' responsive behaviors [8, 16-18]. Those behaviors may include refusing to open their mouth, turning away their head, verbally assaulting the caregiver, spitting at or hitting the caregiver. Responsive behaviors are associated with poor oral health [8, 9].

Depression

O'Neil et al. [19] found an independent, positive association between the number of oral/dental problems and the likelihood of having depression.

References

1. Ong G: **Periodontal disease and tooth loss**. *Int Dent J* 1998, **48**(3 Suppl 1):233-238.
2. Burt BA, Eklund SA: **Dentistry, dental practice, and the community**, 6th edn. St. Louis, MO: Elsevier/Saunders; 2005.
3. Emami E, de Souza RF, Kabawat M, Feine JS: **The impact of edentulism on oral and general health**. *Int J Dent* 2013, **2013**:498305.
4. Preshaw PM, Walls AW, Jakubovics NS, Moynihan PJ, Jepson NJ, Loewy Z: **Association of removable partial denture use with oral and systemic health**. *J Dent* 2011, **39**(11):711-719.
5. Gonda T, MacEntee MI, Kiyak HA, Persson GR, Persson RE, Wyatt C: **Predictors of multiple tooth loss among socioculturally diverse elderly subjects**. *Int J Prosthodont* 2013, **26**(2):127-134.
6. Gerritsen A, Allen PF, Witter D, Bronkhorst E, Creugers N: **Tooth loss and oral health-related quality of life: a systematic review and meta-analysis**. *Health and Quality of Life Outcomes* 2010, **8**(1):126.
7. Polzer I, Schimmel M, Muller F, Biffar R: **Edentulism as part of the general health problems of elderly adults**. *Int Dent J* 2010, **60**(3):143-155.
8. Mancini M, Grappasonni I, Scuri S, Amenta F: **Oral health in Alzheimer's disease: a review**. *Curr Alzheimer Res* 2010, **7**(4):368-373.
9. Noble JM, Scarmeas N, Papapanou PN: **Poor oral health as a chronic, potentially modifiable dementia risk factor: review of the literature**. *Curr Neurol Neurosci Rep* 2013, **13**(10):384.
10. Yao CS, MacEntee MI: **Inequity in oral health care for elderly Canadians: part 1. Oral health status**. *J Can Dent Assoc* 2013, **79**:d114.
11. Friedman PK, Kaufman LB, Karpas SL: **Oral health disparity in older adults: dental decay and tooth loss**. *Dent Clin North Am* 2014, **58**(4):757-770.
12. Martinez-Mier EA, Zandona AF: **The impact of gender on caries prevalence and risk assessment**. *Dent Clin North Am* 2013, **57**(2):301-315.
13. Niessen LC, Gibson G, Kinnunen TH: **Women's oral health: Why sex and gender matter**. *Dent Clin North Am* 2013, **57**(2):181-194.
14. Russell SL, Gordon S, Lukacs JR, Kaste LM: **Sex/Gender differences in tooth loss and edentulism: historical perspectives, biological factors, and sociologic reasons**. *Dent Clin North Am* 2013, **57**(2):317-337.
15. Johnson VB: **Evidence-based practice guideline: oral hygiene care for functionally dependent and cognitively impaired older adults**. *J Gerontol Nurs* 2012, **38**(11):11-19.
16. Chalmers J, Pearson A: **Oral hygiene care for residents with dementia: a literature review**. *J Adv Nurs* 2005, **52**(4):410-419.
17. Jablonski RA, Therrien B, Kolanowski A: **No more fighting and biting during mouth care: applying the theoretical constructs of threat perception to clinical practice**. *Res Theory Nurs Pract* 2011, **25**(3):163-175.
18. Wardh I, Jonsson M, Wikstrom M: **Attitudes to and knowledge about oral health care among nursing home personnel--an area in need of improvement**. *Gerodontology* 2012, **29**(2):e787-792.
19. O'Neil A, Berk M, Venugopal K, Kim SW, Williams LJ, Jacka FN: **The association between poor dental health and depression: findings from a large-scale, population-based study (the NHANES study)**. *Gen Hosp Psychiatry* 2014, **36**(3):266-270.
