# Supplementary material for: Oral/dental items in the resident assessment instrument – minimum Data Set 2.0 lack validity: results of a retrospective, longitudinal validation study
Source: Popul Health Metr. 2016 Oct 21;14:36. doi: 10.1186/s12963-016-0108-y (PMC5073836; doi:10.1186/s12963-016-0108-y)
Supplement: Additional file 2: — Comparison of baseline outcomes between residents with exactly two assessments and residents with three or more assessments. (PDF 16 kb) [file 12963_2016_108_MOESM2_ESM.pdf]

ADDITIONAL FILE 2

Comparison of baseline outcomes between residents with exactly two assessments and residents with three or more assessments

	2 Assessments		3+ Assessments		
	**N**	**%**	**N**	**%**	**P**
Oral/dental problems	69	4.7%	60	4.8%	0.928[A]
Dentate	418	28.4%	336	27.1%	
Dentures	831	56.5%	671	54.1%	0.059[A]
No Dentures	222	15.1%	233	18.8%	
Dementia diagnosis	879	59.8%	727	58.6%	0.556[A]
Debris	163	11.1%	121	9.8%	0.285[A]
No daily cleaning	86	5.8%	85	6.9%	0.303[A]
Female	962	65.4%	887	71.5%	**0.001[A]**
CPS score > 3	357	24.3%	294	23.7%	0.752[A]
ADL-H score > 3	519	35.3%	348	28.1%	**<0.001[A]**
Resists care	432	29.4%	310	25.0%	**0.012[A]**
DRS score > 2	391	26.6%	282	22.7%	**0.023[A]**
	**Mean**	**SD**	**Mean**	**SD**	
Age at assessment	84.68	8.88	84.03	8.83	0.056[B]
Assessment quarter	04/2008	--	03/2009	--	**<0.001[B]**

[A]Two-tailed non-parametric exact test for two independent samples

[B]Two-tailed t-test for two independent samples
