# Supplementary material for: Oral/dental items in the resident assessment instrument – minimum Data Set 2.0 lack validity: results of a retrospective, longitudinal validation study
Source: Popul Health Metr. 2016 Oct 21;14:36. doi: 10.1186/s12963-016-0108-y (PMC5073836; doi:10.1186/s12963-016-0108-y)
Supplement: Additional file 3: — Results of the multicollinearity assessment. (PDF 191 kb) [file 12963_2016_108_MOESM3_ESM.pdf]

ADDITIONAL FILE 3
Results of the multicollinearity assessment

Dependent Variable	Assessment 2	Assessment 3	Assessment 4	Assessment 5	Assessment 6	Dentures	No Dentures	Dementia	Debris	Daily Cleaning
**Independent Variables**	**Tolerance**	**Tolerance**	**Tolerance**	**Tolerance**	**Tolerance**	**Tolerance**	**Tolerance**	**Tolerance**	**Tolerance**	**Tolerance**
**Assessment 2**		0.894	0.804	0.722	0.665	0.664	0.664	0.662	0.661	0.662
**Assessment 3**	0.864		0.760	0.694	0.643	0.642	0.643	0.639	0.639	0.640
**Assessment 4**	0.850	0.833		0.747	0.703	0.703	0.703	0.700	0.699	0.701
**Assessment 5**	0.909	0.905	0.890		0.835	0.834	0.836	0.833	0.833	0.834
**Assessment 6**	0.991	0.991	0.990	0.988		0.985	0.986	0.985	0.985	0.985
**Dentures**	0.618	0.619	0.618	0.616	0.615		0.968	0.615	0.615	0.615
**No Dentures**	0.623	0.625	0.624	0.623	0.621	0.977		0.621	0.621	0.622
**Dementia**	0.875	0.874	0.874	0.874	0.874	0.874	0.874		0.875	0.874
**Debris**	0.961	0.961	0.961	0.961	0.961	0.961	0.962	0.963		0.961
**Daily Cleaning**	0.991	0.991	0.990	0.990	0.989	0.989	0.991	0.989	0.989	
**Female**	0.941	0.942	0.943	0.943	0.941	0.942	0.941	0.943	0.943	0.941
**Age at Assessment**	0.923	0.924	0.923	0.922	0.922	0.936	0.923	0.936	0.922	0.922
**CPS > 3**	0.768	0.769	0.770	0.769	0.767	0.766	0.767	0.809	0.767	0.766
**ALD-H > 3**	0.858	0.859	0.860	0.859	0.857	0.859	0.857	0.859	0.866	0.857
**Resists Care**	0.791	0.791	0.791	0.791	0.791	0.791	0.792	0.811	0.794	0.791
**DRS Score > 2**	0.873	0.872	0.871	0.871	0.871	0.871	0.871	0.871	0.877	0.871
**Diabetes Mellitus**	0.962	0.962	0.962	0.962	0.962	0.963	0.962	0.963	0.962	0.962
**Atherosclerotic Heart Disease**	0.985	0.985	0.985	0.986	0.985	0.985	0.985	0.985	0.985	0.985
**Pneumonia**	0.995	0.995	0.995	0.995	0.995	0.995	0.995	0.995	0.995	0.995
**Quarter of Assessment**	0.756	0.808	0.807	0.739	0.660	0.652	0.653	0.653	0.652	0.653

CPS = Cognitive Performance Scale, ADL-H = Activities of Daily Living - Hierarchy Scale, DRS = Depression Rating Scale

Dependent Variable	Female	Age at Assessment	CPS Score > 3	ADL-H Score > 3	Resists Care	DRS Score > 2	Diabetes Mellitus	Atherosclerotic Heart Disease	Pneumonia	Quarter of Assessment
**Independent Variables**	**Tolerance**	**Tolerance**	**Tolerance**	**Tolerance**	**Tolerance**	**Tolerance**	**Tolerance**	**Tolerance**	**Tolerance**	**Tolerance**
**Assessment 2**	0.661	0.662	0.663	0.662	0.661	0.663	0.661	0.662	0.661	0.767
**Assessment 3**	0.639	0.640	0.641	0.640	0.639	0.639	0.639	0.639	0.639	0.790
**Assessment 4**	0.701	0.701	0.703	0.701	0.699	0.699	0.699	0.700	0.699	0.865
**Assessment 5**	0.835	0.833	0.836	0.834	0.833	0.833	0.833	0.834	0.833	0.944
**Assessment 6**	0.985	0.985	0.986	0.985	0.985	0.985	0.985	0.985	0.985	0.996
**Dentures**	0.616	0.625	0.615	0.616	0.615	0.616	0.616	0.615	0.615	0.615
**No Dentures**	0.621	0.622	0.621	0.621	0.621	0.621	0.621	0.621	0.621	0.621
**Dementia**	0.876	0.887	0.922	0.876	0.896	0.874	0.875	0.874	0.874	0.874
**Debris**	0.963	0.962	0.963	0.971	0.965	0.968	0.961	0.962	0.961	0.961
**Daily Cleaning**	0.989	0.989	0.989	0.989	0.989	0.990	0.989	0.989	0.989	0.989
**Female**		0.954	0.942	0.949	0.953	0.943	0.945	0.943	0.943	0.943
**Age at Assessment**	0.934		0.926	0.922	0.922	0.922	0.937	0.924	0.922	0.922
**CPS Score > 3**	0.767	0.770		0.838	0.801	0.767	0.769	0.766	0.766	0.767
**ALD-H Score > 3**	0.865	0.857	0.938		0.858	0.858	0.857	0.857	0.858	0.858
**Resists Care**	0.801	0.791	0.827	0.791		0.865	0.792	0.791	0.791	0.792
**DRS Score > 2**	0.873	0.871	0.872	0.872	0.953		0.872	0.873	0.871	0.874
**Diabetes Mellitus**	0.966	0.978	0.965	0.962	0.963	0.964		0.966	0.962	0.963
**Atherosclerotic Heart Disease**	0.987	0.987	0.985	0.985	0.985	0.988	0.989		0.985	0.985
**Pneumonia**	0.997	0.995	0.995	0.996	0.995	0.995	0.995	0.995		0.995
**Quarter of Assessment**	0.654	0.652	0.653	0.653	0.653	0.655	0.653	0.652	0.653	

CPS = Cognitive Performance Scale, ADL-H = Activities of Daily Living - Hierarchy Scale, DRS = Depression Rating Scale
