# Supplementary material for: Oral/dental items in the resident assessment instrument – minimum Data Set 2.0 lack validity: results of a retrospective, longitudinal validation study
Source: Popul Health Metr. 2016 Oct 21;14:36. doi: 10.1186/s12963-016-0108-y (PMC5073836; doi:10.1186/s12963-016-0108-y)
Supplement: Additional file 4: — Results of the binary logistic regression models. (PDF 36 kb) [file 12963_2016_108_MOESM4_ESM.pdf]

ADDITIONAL FILE 4
Results of the binary logistic regression models

	Assessment 1 (n=2711)			Assessment 2 (n=2711)			Assessment 3 (n=1240)			Assessment 4 (n=537)			Excluded admission assessments (n=5132)		
**Parameter**	**Est.**	**95% CI**	**P**	**Est.**	**95% CI**	**P**	**Est.**	**95% CI**	**P**	**Est.**	**95% CI**	**P**	**Est.**	**95% CI**	**P**
Dentate		Reference			Reference			Reference			Reference			Reference	
Dentures	0.665	0.412-1.074	0.096	**0.427**	**0.257-0.710**	**0.001**	**0.232**	**0.110-0.490**	**0.000**	0.348	0.084-1.441	0.145	**0.600**	**0.429-0.839**	**0.003**
No Dentures	**3.290**	**2.080-5.206**	**<0.001**	**2.661**	**1.685-4.203**	**<0.001**	1.692	0.894-3.202	0.106	2.853	0.778-10.458	0.114	**2.853**	**2.101-3.874**	**0.000**
Dementia diagnosis	0.841	0.572-1.236	0.379	0.929	0.619-1.393	0.720	0.868	0.487-1.549	0.632	2.633	0.787-8.804	0.116	1.095	0.831-1.442	0.519
Debris	**2.454**	**1.533-3.931**	**<0.001**	**2.390**	**1.555-3.674**	**<0.001**	1.319	0.664-2.621	0.430	**3.018**	**1.089-8.367**	**0.034**	**2.500**	**1.893-3.301**	**0.000**
Daily cleaning	0.980	0.481-1.995	0.955	1.366	0.484-3.852	0.556	1.149	0.251-5.266	0.858	0.864	0.097-7.722	0.896	0.863	0.487-1.529	0.613
female	0.739	0.508-1.075	0.113	0.827	0.572-1.196	0.313	0.859	0.496-1.486	0.586	1.705	0.562-5.169	0.346	**0.671**	**0.522-0.862**	**0.002**
Age at assessment	0.983	0.965-1.002	0.080	**0.979**	**0.962-0.996**	**0.018**	0.986	0.961-1.011	0.268	0.990	0.941-1.041	0.682	1.004	0.990-1.018	0.608
CPS score > 3	0.696	0.432-1.122	0.137	0.970	0.635-1.482	0.889	0.740	0.412-1.327	0.312	0.943	0.368-2.417	0.903	1.073	0.806-1.429	0.630
ADL-H score > 3	0.744	0.489-1.132	0.167	1.187	0.806-1.748	0.385	**2.012**	**1.179-3.434**	**0.010**	1.173	0.466-2.952	0.735	**1.388**	**1.073-1.795**	**0.012**
Resists care	1.405	0.912-2.163	0.123	1.266	0.846-1.895	0.252	1.222	0.691-2.161	0.491	0.675	0.276-1.653	0.390	0.984	0.739-1.309	0.911
DRS score > 2	0.952	0.611-1.485	0.829	1.278	0.874-1.868	0.206	0.932	0.542-1.603	0.800	1.053	0.448-2.476	0.906	**1.562**	**1.191-2.049**	**0.001**
Assessment quarter	0.991	0.951-1.032	0.659	1.038	0.998-1.081	0.063	0.996	0.922-1.075	0.909	**1.258**	**1.040-1.522**	**0.018**	1.010	0.991-1.030	0.313

CPS = Cognitive Performance Scale, ADL-H = Activities of Daily Living - Hierarchy Scale, DRS = Depression Rating Scale
