# Supplementary material for: Oral/dental items in the resident assessment instrument – minimum Data Set 2.0 lack validity: results of a retrospective, longitudinal validation study
Source: Popul Health Metr. 2016 Oct 21;14:36. doi: 10.1186/s12963-016-0108-y (PMC5073836; doi:10.1186/s12963-016-0108-y)
Supplement: Additional file 5: — Additional General Estimating Equations (final, fully adjusted model), using variables of a) the assessment conducted previously to the assessment including the oral/dental issues variable and b) the admission assessment. (PDF 19 kb) [file 12963_2016_108_MOESM5_ESM.pdf]

ADDITIONAL FILE 5

Additional General Estimating Equations (final, fully adjusted model), using variables of a) the assessment conducted previously to the assessment including the oral/dental issues variable and b) the admission assessment

	Previous Assessment			Admission Assessment		
**Parameter**	**Est.**	**95% CI**	**P**	**Est.**	**95% CI**	**P**
Assessment 2	Reference			Reference		
Assessment 3	0.987	0.741-1.313	0.926	1.167	0.906-1.504	0.231
Assessment 4	0.871	0.554-1.369	0.549	1.156	0.762-1.753	0.495
Assessment 5	0.561	0.251-1.255	0.159	1.090	0.518-2.292	0.821
Assessment 6	1.428	0.220-9.260	0.709	2.361	0.281-19.843	0.429
Dentate	Reference			Reference		
Dentures	**0.347**	**0.217-0.554**	**0.000**	**0.404**	**0.273-0.599**	**0.000**
No Dentures	**2.319**	**1.492-3.606**	**0.000**	**1.828**	**1.234-2.707**	**0.003**
Dementia diagnosis	1.038	0.705-1.528	0.849	0.994	0.701-1.411	0.975
Debris	**2.122**	**1.447-3.11**	**0.000**	**1.870**	**1.202-2.910**	**0.005**
Daily cleaning	1.276	0.591-2.757	0.534	0.764	0.451-1.295	0.318
Female	0.949	0.673-1.339	0.767	0.943	0.670-1.327	0.736
Age at assessment	**0.983**	**0.967-0.999**	**0.040**	**0.981**	**0.965-0.997**	**0.024**
CPS score > 3	0.869	0.600-1.258	0.458	1.065	0.729-1.555	0.744
ADL-H score > 3	**1.434**	**1.020-2.016**	**0.038**	0.877	0.598-1.284	0.499
Resists care	1.159	0.840-1.600	0.369	1.289	0.883-1.881	0.189
DRS score > 2	1.175	0.859-1.608	0.313	1.212	0.823-1.784	0.330
Assessment quarter	1.033	0.993-1.074	0.108	1.033	0.993-1.075	0.111

CPS = Cognitive Performance Scale, ADL-H = Activities of Daily Living - Hierarchy Scale, DRS = Depression Rating Scale
